# Epithelial ovarian cancer: A case report

**DOI:** 10.3892/etm.2014.1970

**Published:** 2014-09-17

**Authors:** JIANYUAN GAO, HAIYING FANG, XIAOMING WANG, LIPING WU, RONGHUAI ZHANG, YAJUN HAN

**Affiliations:** Department of the VIP Ward, The First Affiliated Hospital, The Fourth Military Medical University of the PLA, Xi’an, Shaanxi 710032, P.R. China

**Keywords:** recurrent epithelial ovarian cancer, bevacizumab, carboplatin, chemotherapy, massive pleural effusions

## Abstract

An 82-year-old female was diagnosed with ovarian cancer in May 2004. Following gynecological surgery, pathological evaluation showed stage IIIC epithelial ovarian cancer. From June 2004 to January 2005, the patient received six cycles of conventional treatment combined with intravenous paclitaxel (Taxol^®^) and cisplatin. The patient developed abdominal distension and experienced a gradual deterioration in health during 2007, with admission to The First Affiliated Hospital in May 2007. The patient presented with severe abdominal distension and breathing difficulty on May 15 and appeared to be in critical condition. Ultrasound examination revealed massive ascites and left-side pleural effusion. Thoracentesis and abdominocentesis were performed, and 300 mg carboplatin was administered intraperitoneally on May 19, followed by a second abdominocentesis on May 21. However, these treatments did not alleviate the symptoms, and 200 mg bevacizumab was administered by intravenous infusion on May 27. The condition of the patient gradually improved and 400 mg bevacizumab was administered by intravenous infusion every two weeks from June 9. From December, the dosage of bevacizumab was reduced to 200 mg every two weeks. In addition, 300 mg carboplatin was administered intraperitoneally on November 4 and intraperitoneal carboplatin chemotherapy was repeated thereafter. The patient exhibited disease-free survival until July 2009, at which time disease progression was observed and the cancer recurred in August 2009. The patient died of multiple organ failure in September 2009. Bevacizumab rapidly eliminated the patient’s massive ascites and pleural effusion, and achieved an effect that was not possible with other treatments. Therefore, bevacizumab is an effective therapy for late-stage relapse and refractory ovarian cancer.

## Introduction

Ovarian epithelial carcinoma is one of the most common gynecological cancers (1). Once clinical symptoms are apparent, patients have developed mid- to late-stage ovarian cancer, at which point aggressive treatments may yield more favorable results than conservative ones. Surgery remains the primary treatment for stage III ovarian cancer and allows removal of gross disease. Following surgery, intravenous (IV) or intraperitoneal (IP) chemotherapy extends the survival time of patients and is associated with superior results for recurrent ovarian cancer relative to other treatment modes. In addition to IV and IP chemotherapy, other treatment regimens, including oral chemotherapy, hormones and gonadotrophin-releasing hormone antagonists, exert certain positive effects (2). More recently, biologic therapy for ovarian cancer has become an area of focus and may be attempted in clinical treatment (3).

Patients treated with IP paclitaxel/cisplatin chemotherapy have been observed to live significantly longer lives than those treated with IV paclitaxel/cisplatin chemotherapy, which indicates that IP administration is the optimal route for ovarian cancer treatment (4). Paclitaxel/carboplatin chemotherapy is more suitable for patients with a poor performance status than for patients with a good performance status. However, the outcome of chemotherapy for elderly patients with advanced disease and a poor performance status has not been reported. Chemotherapy is also contraindicated for patients with Eastern Cooperative Oncology Group (ECOG) performance statuses of 3 or 4.

The combination of bevacizumab with carboplatin/paclitaxel is associated with significantly longer disease-free survival than carboplatin/paclitaxel alone. However, no significant difference has been observed between bevacizumab plus carboplatin/paclitaxel and carboplatin/paclitaxel in terms of overall survival rate (5). Thus, bevacizumab does not appear to confer any survival advantage, and the National Comprehensive Cancer Network panel does not recommend its use. Bevacizumab may be used as a maintenance drug subsequent to chemotherapy; however, clinical studies have indicated that it results in numerous many side effects. Therefore, clinicians should consider the advantages and disadvantages of bevacizumab prior to prescribing the drug to patients.

## Case report

An 82-year-old female, who presented with abdominal distension, abdominal pain, a slight sense of fullness subsequent to eating and increased urination frequency every day for two months was diagnosed with ovarian cancer in May 2004. The attending gynecologist performed a hysterectomy, a bilateral salpingo-oophorectomy and an omentectomy, and removed all evidence of gross disease. Diffuse metastatic involvement of the omentum and diaphragmatic metastasis were observed. Pathological assessment showed stage IIIC epithelial ovarian cancer. From June 2004 to January 2005, the patient received six cycles of conventional treatment in combination with IV paclitaxel (Bristol-Myers Squibb Co., Princeton, New Jersey, USA) and cisplatin (Qilu Pharmaceutical Co., Ltd, Jinan, China). Following surgery and chemotherapy, the patient’s cancer antigen-125 (CA-125) levels declined to 7.3 U/ml. The patient’s abdominal distension and pain, and the sense of fullness subsequent to eating were alleviated, while the urination frequency was reduced. Furthermore, the patient’s appetite improved significantly.

In 2007, the patient developed abdominal distension and gradually deteriorated, and was admitted to The First Affiliated Hospital, The Fourth Military Medical University of the PLA (Xi’an, China) on May 8, 2007. Physical examination and laboratory analyses revealed dry and moist rales in both lungs, shifting dullness in the abdomen and a CA-125 level of 1,030 U/ml. Computed tomography (CT) scans (Model 5124069-5; GE Healthcare Co. Milwaukee, WI, USA) revealed an infection in the right upper lobe of the right lung, left-sided pleural effusion, sclerotic lesions in the apex of the right lung and the lower lobe of the left lung and a little ascites. Hypodense lesions in the right lobe of the liver and spleen and enlarged retroperitoneal lymph nodes were considered metastatic. Pelvic CT scans showed postoperative agenesis of the uterus and ovaries, as well as soft tissue shadows at the junction between the rectum and sigmoid colon, and in front of the intestine. The condition of the patient gradually deteriorated; she was immobile, with an ECOG performance status of 4 and further chemotherapy was not considered. Instead, doses of 50 mg etoposide (Jiangsu Hengrui Medicine Co., Ltd. Lianyungang, China) and 40 mg megestrol acetate (Bristol-Myers Squibb Co.) were administered orally once daily. On May 12, 3.6 mg goserelin acetate (AstraZeneca UK Limited, Cheshire, UK) implant was injected subcutaneously.

The patient’s condition continued to decline with the development of severe abdominal distension, breathing difficulties and an inability to eat on 15 May. Ultrasound (iU33; Philips Ultrasound, Bothell, WA, USA) showed massive ascites and left pleural effusion, and the patient appeared to be in a critical condition. Treatment with etoposide and megestrol acetate was discontinued and parenteral nutrition was provided. The patient underwent thoracentesis and abdominocentesis, during which 1,000 ml clear yellow ascitic fluid was extracted, and 300 mg carboplatin (Qilu Pharmaceutical Co., Ltd) was injected intraperitoneally into the abdominal cavity on May 19. The patient’s condition did not improve, and 1,500 ml clear yellow ascite fluid was removed on May 21. The condition of the patient remained poor until May 27, at which time, an IV infusion of 200 mg bevacizumab (Avastin^®^, Genentech, Inc., South San Francisco, CA, USA) was administered. Written informed consent was obtained from the patient. Thoracentesis was performed again when the patient complained of breathing difficulties, and 1,500 ml clear yellow pleural fluid was extracted on May 31.

**Figure 1:**
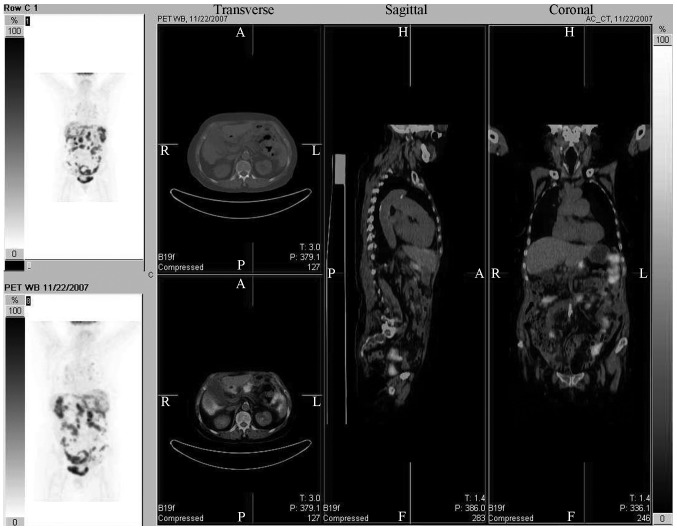
Positron emission tomography and computed tomography scans showed widespread metastases in the body of the patient on November 22, 2007.

From June 1, 2007, a gradual improvement was observed in the patient’s condition. Dyspnea, abdominal distension and abdominal pain were significantly reduced, the patient had a significant improvement in appetite and urine and feces had returned to normal. The patient was upright and mobile. Bevacizumab was administered by IV infusion at a dosage of 400 mg every two weeks from June 9, followed by a dosage of 200 mg every two weeks from the end of November. On June 10, 2007, ultrasound showed that the massive left-side pleural effusion had disappeared. On June 29, 2007, a CT scan showed that the ascites, and the liver, spleen and pelvic lesions had disappeared. The patient’s CA-125 level was 31.17 U/ml on July 3, 2007, and 300 mg IP carboplatin was administered on November 4, 2007. Positron emission tomography-CT scans (Biograph^™^ TruePoint^™^ PET•CT; Siemens Medical Solutions USA, Inc., Knoxville, TN, USA) showed widespread metastases in the patient’s body on November 22, 2007 (Fig. 1). IP carboplatin was administered several times. The patient exhibited disease-free survival until July 2009 when disease progression was observed, followed by disease recurrence in August 2009. The patient died of multiple organ failure in September 2009.

## Discussion

Following ovarian cancer surgery, the patient exhibited disease-free survival for several years until the cancer recurred and a CT scan revealed widespread intra-abdominal metastases. The patient had concomitant diseases and developed massive ascites and pleural effusion. Treatment included IP chemotherapy and oral etoposide. These treatments were ineffective and the condition of the patient deteriorated further. To the best of our knowledge, no similar clinical case has been reported, and palliative care remains the recommended treatment for such cases (2,6).

Ascites disappear rapidly with bevacizumab treatment (7–9). In the present case study, massive effusion in the thoracic and abdominal cavity disappeared within a short time-frame subsequent to bevacizumab administration, thereby providing a basis for the next treatment. Bevacizumab was administered by IV infusion every two weeks and IP carboplatin was administered intermittently until the death of the patient. The patient was likely to have died earlier if pleural effusion had not been rapidly controlled; by contrast, the patient experienced disease-free survival for 25 months and treatment was associated with favorable results. Thus, bevacizumab may be concluded to play a key role in disease control.

Bevacizumab in combination with paclitaxel and carboplatin improves the progression-free survival of patients with ovarian cancer; however, it does not increase their overall survival time. Furthermore, the use of the drug in maintenance therapy subsequent to chemotherapy does not appear to increase overall survival (5). Other studies have indicated that bevacizumab does not increase overall survival in any cancer type, despite its ability to prolong progression-free survival (10–12). Thus, it was concluded that no overall survival benefit may be derived from bevacizumab. However, chemotherapy is contraindicated for patients with massive ascites, massive pleural effusions, breathing difficulties and poor performance status. Continuing chemotherapy in such cases may aggravate the patient’s condition and possibly lead to death. In the present case study, the patient’s ascites and pleural effusion disappeared following administration of bevacizumab, and a significant improvement in performance status was observed. IP chemotherapy combined with bevacizumab also led to extended disease-free survival. Thus, the role of bevacizumab in prolonging survival in patients with ovarian cancer warrants further study. The pulmonary artery systolic pressure of the patient rose continually until her death; such increases in arterial pressure may be associated with the use of bevacizumab.

Chemotherapy regimens for platinum-sensitive relapsed ovarian cancer include monotherapy with agents such as cisplatin and carboplatin, as well as combination therapies, including paclitaxel plus carboplatin, carboplatin plus gemcitabine and cisplatin plus gemcitabine (13–16). Other regimens may also be effective, including monotherapy with paclitaxel, docetaxel, cyclophosphamide, ifosfamide, oxaliplatin, melphalan, irinotecan, capecitabine, pemetrexed and etoposide, and hormone therapy with tamoxifen, anastrozole, letrozole, leuprorelin acetate and megestrol acetate (16–18). In the present case study, the patient received goserelin acetate implant, etoposide and megestrol acetate; however, these treatments were not efficacious. IP carboplatin was beneficial until the patient was no longer able to tolerate the treatment. Carboplatin was prescribed as it has a lower impact on the kidney than other drugs. This treatment strategy had a favorable outcome for the patient.

Bevacizumab rapidly eliminated the massive ascites and pleural effusion and achieved an effect that was not able to be attained with other treatments. Bevacizumab appears to be an effective means of controlling late-stage relapse and refractory ovarian cancer. Future clinical studies are required to confirm the effectiveness of the drug.
